# Participation of preovulatory follicles in the activation of primordial follicles in mouse ovaries

**DOI:** 10.7150/ijbs.95020

**Published:** 2024-07-08

**Authors:** Jingwen Zhang, Wenzhe Xia, Jiaqi Zhou, Shaogang Qin, Lin Lin, Ting Zhao, Huarong Wang, Chen Mi, Yifan Hu, Zixuan Chen, Tianhua Zhu, Xinyu Yang, Tuo Zhang, Guoliang Xia, Yuwen Ke, Chao Wang

**Affiliations:** 1 State Key Laboratory of Animal Biotech Breeding, College of Biological Sciences, China Agricultural University, Beijing 100193, China.; 2 School of Life Sciences and Medicine, Shandong University of Technology, Shandong 255049, China.; 3 Department of Obstetrics and Gynecology, Key Laboratory for Major Obstetric Diseases of Guangdong Province, The Third Affiliated Hospital of Guangzhou Medical University, Guangzhou 510000, China.; 4 Key Laboratory of Animal Ecology and Conservation Biology, Institute of Zoology, Chinese Academy of Sciences, Beijing 100101, China.; 5 Transformation Engineering Research Center of Chronic Disease Diagnosis and Treatment, Department of Physiology, College of Basic Medicine, Guizhou Medical University, Guiyang, Guizhou, 550025, China.

**Keywords:** Ovulation, primordial follicle activation, extracellular matrix, Cathepsin L.

## Abstract

The mechanisms behind the selection and initial recruitment of primordial follicles (PmFs) from the non-growing PmF pool during each estrous cycle in females remain largely unknown. This study demonstrates that PmFs closest to the ovulatory follicle are preferentially activated in mouse ovaries under physiological conditions. PmFs located within 40 μm of the ovulatory follicles were more likely to be activated compared to those situated further away during the peri-ovulation period. Repeated superovulation treatments accelerated the depletion of the PmF reserve, whereas continuous suppression of ovulation delayed PmF reserve consumption. Spatial transcriptome sequencing of peri-ovulatory follicles revealed that ovulation primarily induces the degradation and remodeling of the extracellular matrix (ECM). This ECM degradation reduces mechanical stress around PmFs, thereby triggering their activation. Specifically, Cathepsin L (CTSL), a cysteine proteinase and lysosomal enzyme involved in ECM degradation, initiates the activation of PmFs adjacent to ovulatory follicles in a distance-dependent manner. These findings highlight the link between ovulation and selective PmF activation, and underscore the role of CTSL in this process under physiological conditions.

## 1. Introduction

In female mammals, the ovarian follicle serves as the fundamental structural and functional unit of reproduction. A primordial follicle (PmF) consists of a non-growing oocyte arrested at the diplotene stage of the first meiosis, which remains transcriptionally and metabolically active but distinct from the growing oocytes in primary follicles. This oocyte is surrounded by several peripheral monolayer flat precursor granulosa cells (preGCs) 1, 2. The pool of PmFs, established around the time of birth, serves as the sole source of germ cells throughout a female's lifetime 3-6. Consequently, female fertility is heavily dependent on the PmF reserve within the ovaries. While most PmFs remain in a non-growing state 7, mature oocyte production begins with the cyclic activation of PmF cohorts 1. These PmFs gradually develop into primary and secondary follicles, with some eventually responding to gonadotropins such as follicle-stimulating hormone (FSH) and luteinizing hormone (LH). Each estrous cycle's ovulation is triggered by the LH surge, a principle that guides the collection of high-quality mature oocytes for assisted reproduction practices.

However, the selective activation of PmFs during ovarian development, which may involve various mechanisms, remains poorly understood. This knowledge gap hinders a comprehensive understanding of premature ovarian insufficiency (POI), affecting 1%-2% of women 8, and limits the development of effective treatments for POI, characterized by the cessation of menstrual cycles before age 40.

Recent studies have begun to elucidate the mechanisms behind PmF activation. Research using gene depletion mouse models has identified the mechanistic target of rapamycin complex 1 (mTORC1) signaling pathway in preGCs as a key initiator of PmF activation. This pathway responds to nutritional, mechanical, oxygen, or energy cues in the niche 9-11. PreGCs differentiate into granulosa cells (GCs), proliferate, and secrete the paracrine factor KIT ligand (KITL), which activates the PI3K (phosphatidylinositol 3-kinase) signaling pathway in oocytes by binding to the KIT receptor on the oocyte membrane 12. The cytoplasmic localization of Forkhead box O3 (FOXO3a) protein is also a critical marker for PmF activation 13-15. In oocytes, FOXO3a shuttles between the nucleus and cytoplasm, importing into the nucleus during PmF assembly and exporting upon PmF activation. Oocyte-specific ablation of PTEN, which counteracts PI3K, leads to PI3K-induced Akt activation and FOXO3a hyperphosphorylation. Once phosphorylated FOXO3a exits the nucleus, the PmF is activated and begins to grow 16-18. Based on these discoveries, *in vitro* activation (IVA) of PmFs targeting key mTOR/PI3K pathway proteins has enabled the birth of babies in Asian countries over the past decade 19. Additionally, the activation of the first wave of follicles in prepubertal mice, originating from germ cells before follicle formation, appears to be KITL-independent and initiated by the oocyte 2. However, existing research provides limited insight into why only a subset of PmFs are selectively activated after puberty *in vivo*.

Localized trauma induced by ovarian interventions may accelerate PmF activation and follicle growth. Earlier studies have shown that ovarian procedures such as cystectomy, wedge resection, and laser drilling therapies for polycystic ovarian syndrome (PCOS), which affects 5%-10% of infertile women, improve general follicular development 20-22. A decade ago, it was discovered that fragmentation of murine ovaries resulted in a reduced number of PmFs, improved follicle growth, and the generation of mature oocytes 21. This effect is believed to be achieved by promoting actin polymerization, disrupting ovarian Hippo signaling, and increasing the expression of downstream growth factors 21. Later, He *et al*. found that after partial ovarian resection surgery, a localized increase of nerve growth factor (NGF) in the ovarian mesenchyme activated PmFs by functioning upstream of mTOR signaling 20. These studies provide valuable insights into PmF activation but are limited to pathological or surgical conditions rather than physiological ones.

Ovulation, driven by LH, resembles an inflammatory response 23, 24. During ovulation, the degradation of the ovarian cortical mesenchymal extracellular matrix (ECM) at the follicle apex facilitates cumulus oocyte complex (COC) release 25. Recent studies have shown that the ECM is crucial for maintaining the non-growing state of PmFs by binding actin and collagen fibers to produce mechanical stress, which influences the rotation of the oocyte nucleus within a PmF 26. PmFs become activated once the surrounding ECM is digested 26. Notably, consecutive superovulation (CSO) treatment reduces the PmF reserve by decreasing the number of PmFs, inducing atresia 27, or causing POI-like symptoms 28. This results in decreased ratios of follicles at all developmental stages and severely impaired oocyte and embryo quality compared to controls 29-33. Given that ovulation itself may induce localized trauma and ECM degradation around the ovulation site, it is speculated that ovulation may have unidentified effects on the activation of surrounding PmFs, potentially key to selective PmF activation under physiological conditions.

This study found that PmFs within 40 μm of ovulating follicles in adult mouse ovaries were more likely to be activated than those located farther away. Spatial transcriptome sequencing analysis and *in vitro* assays revealed that high levels of Cathepsin L (CTSL), a cysteine proteinase, were specifically enriched around ovulating follicles. CTSL degraded the ECM, reduced mechanical stress around the PmF, and eventually led to PmF activation. In summary, PmFs adjacent to ovulatory follicles may have a priority for orderly and selective activation during the natural estrous cycle in adult females. These findings contribute to understanding the mechanisms of PmF activation adjacent to ovulating follicles via ECM degradation by CTSL.

## 2. Materials and methods

### 2.1 Animals

Adult CD-1 mice, aged three weeks and six to eight weeks, were purchased from Beijing Vital River Laboratory Animal Technology Co., Ltd. Additionally, CD-1 mice at seven days postpartum (dpp) were obtained from Beijing HFK Bioscience Co., Ltd. Mice were housed under controlled lighting conditions (12 hours light, 12 hours dark) and maintained a temperature range of 24 °C to 26 °C. The mice had free access to food and water in a Specific Pathogen Free (SPF) grade facility. All animal experiments received approval from the Institutional Animal Care and Use Committee of China Agricultural University (license number: SYXK (Beijing) 2019-0026) and adhered to its standards. All experiments comply with the ethical principles of animal welfare (AW60502202-3-1)

### 2.2 Immunofluorescence

Fresh ovarian tissue was separated under a microscope and fixed overnight in 4% paraformaldehyde, then dehydrated with gradient ethanol and soaked in paraffin wax at 60°C for several hours with xylene transparent tissue. A manually operated rotary microtome was used to cut 5 μm paraffin sections. The tissues were then dewaxed with xylene and a gradient ethanol series. For antigen retrieval, 0.01% sodium citrate buffer (pH 6.0) was used and the following microwave conditions were applied: high power for 4 minutes, medium-high power for 4 minutes, and low power twice for 4 minutes each. The slides were blocked with 10% normal donkey serum for 1 hour at room temperature.

The ovarian tissue was embedded in OCT and freeze-sectioned it at -20 °C for Phalloidin and Collagen IV staining. Slides were incubated with primary antibodies overnight. The primary antibodies and their dilution ratios were as follows: FOXO3a antibody (Cell Signaling Technology, 12829S) (1:200), MVH antibody (Abcam, ab27591) (1:200), PAI-1 antibody (Santa Cruz, sc-166539) (1:50), CTSL antibody (Santa Cruz, sc-390367) (1:50), Phalloidin antibody (ThermoFisher, R415) (1:200), and Collagen IV antibody (Abcam, ab19808) (1:200). Ovarian sections were incubated with Alexa Fluor 488 or Alexa Fluor 555 conjugated secondary antibodies for 1 hour at 37 °C. Subsequently, sections were incubated with Hoechst for 1 hour at 37 °C. Finally, anti-fade fluorescence mounting medium (Ruitaibio) was applied to the ovarian sections and sealed the slides with coverslips. Sections were photographed using Leica THUNDER Imager, Nikon A1, or OLYMPUS SZX16 as needed.

### 2.3 Counting of the activated PmFs

5 μm continuous ovarian sections were stained from mice with FOXO3a and MVH co-immunofluorescence to distinguish activated primordial follicles (PmFs) from non-growing ones, using MVH to specifically label the oocyte cytoplasm. According to John *et al*. 17, an activated PmF is characterized by the co-localization of both FOXO3a and MVH fluorescence in the oocyte cytoplasm. In contrast, a non-growing PmF shows FOXO3a localized in the nucleus and MVH in the cytoplasm. To ensure accurate identification, each follicle was judged by the largest interface of the nucleus. The total number of activated PmFs were calculated by multiplying the number of counted PmFs by 5.

### 2.4 Western blotting

Ovarian tissue was homogenized using WIP solution and PMSF (Cell Signaling Technologies, 8553S) and added it to 1 × SDS-PAGE protein loading buffer (Beyotime, P0015L). The mixture was incubated in a 100 °C water bath for 10 minutes to extract proteins. The protein samples were then subjected to 10% SDS-PAGE gel electrophoresis and transferred to a PVDF membrane (Millipore, ISEQ00005). The membrane was blocked with 5% skim milk powder at room temperature for 1 hour. The membrane was incubated with primary antibodies overnight at 4 °C. The primary antibodies and their respective dilution ratios were as follows: anti-CYP11A1 (Cell Signaling Technology, 14217S) (1:1000), anti-mTOR (Cell Signaling Technologies, 2983S) (1:1000), anti-p-mTOR (Santa Cruz, sc-293133) (1:500), anti-FOXO3a (Cell Signaling Technology, 12829S) (1:1000), anti-p-FOXO3a (Santa Cruz, Sc-12357) (1:500), anti-AKT (Cell Signaling Technology, 4685) (1:1000), anti-p-AKT (Beyotime Biotechnology, AA331) (1:1000), anti-CTSL (Santa Cruz, sc-390385) (1:500), and β-actin (Cwbiotech, CW0096M) (1:1000). The PVDF membrane was washed in TBST three times and then incubated it with a secondary antibody (ZSGB-BIO, 1:5000) at room temperature for 1 hour. Finally, proteins were visualized using a Tanon 5200 imager.

### 2.5 Superovulation and ovulation inhibition

Superovulation Procedure: the superovulation procedure was conducted on 6-8-week-old adult ICR mice, as proestrus and diestrus are the optimal stage of the estrus cycle for superovulation 34. Mice were identified at the proestrus and diestrus stages using vaginal smears and performed superovulation during each reproductive cycle. The selected mice were treated sequentially with equine chorionic gonadotropin (eCG) and human chorionic gonadotropin (hCG) for 10 consecutive estrus cycles.

Ovulation Inhibition Procedure: ovulation inhibition was also studied in 6-8-week-old adult ICR mice. Using vaginal smears, mice were selected at the proestrus and diestrus stages for ovulation inhibition during each reproductive cycle. The selected mice were treated with Ethynodiol diacetate (MCE, HY-B1089) for 10 consecutive estrus cycles. Based on the mice's body weight, drug concentration gradients were established at 30 μg/kg, 50 μg/kg, and 70 μg/kg for the treatments. Initial results indicated that ovulation activity was significantly inhibited at a concentration of 70 μg/kg.

### 2.6 Spatial Transcriptome Sequencing

Ovaries treated with eCG for 46 hours followed by hCG for 12 hours were collected, and frozen slides of 10 μm thickness were prepared (Figure 3D). Slides can be stored in a -80 °C freezer for up to 1 month. The frozen tissue section slide was removed from the -80 °C freezer, placed on an ultra-clean platform to air dry for approximately 10 min, and the OCT on the tissue surface was removed using 1 × PBS with RNase Inhibitor (0.05 U/μL, Enzymatics, Y9240L). The tissue was then fixed with 4% PFA for about 20 min and washed three times with 1 × PBS with RNase Inhibitor. The fixed tissue was permeabilized with 0.05% Triton X-100 (Sigma-Aldrich, T8787) in PBS for 20 min and washed twice with 0.5 × PBS with RNase Inhibitor. Subsequently, the reverse transcription mixture was prepared as follows: 24.9 μL of 30% PEG6000 (Sigma-Aldrich, 81253), 12 μL of 5 × RT buffer, 12 μL of 50 μM RT primer with an affinity tag, 7.5 μL of 200 U/μL Maxima H Minus Reverse Transcriptase (Thermo Fisher, EP0753), 3 μL of 10 mM dNTP (New England Biolabs, N0447S), and 0.6 μL of RNase Inhibitor (Enzymatics). The mixture was incubated for 30 min at room temperature followed by 90 min in a 42 °C wet box. After completion of reverse transcription, NIB buffer (10 mM Tris buffer pH 7.5, 10 mM NaCl, 3 mM MgCl_2_, 0.1% NP-40) with 1 μL of 0.5 M EDTA was added, and the slides were washed once using DEPC water.

The first PDMS microfluidic chip was covered and passed through the tissue region via the chip channel. A barcode mixture (139 μL Nuclease-Free water (not DEPC-Treated, Invitrogen, AM9930), 54 μL of 10 × T4 Ligase Buffer (New England Biolabs, B0202SVIAL), 5.4 μL of 10% Triton X-100 (Sigma-Aldrich, T8787), 22 μL of T4 DNA Ligase (400 U/μL, New England Biolabs, M0202M), 4.4 μL of RNase Inhibitor (Enzymatics), 1 μL of SUPERase In RNase Inhibitor (Invitrogen, AM2696), and 1 μL of a specific barcode (Table S1)) was added. The mixture was incubated at 37 °C for 30 min. Blocking A was then added to each microwell, followed by 1 × NEB buffer 3.1 (New England Biolabs, B6003S) to wash the channel. The PDMS microfluidic chip was evacuated, removed, and the slides were washed again with DEPC water. The second round of PDMS microfluidic chip was operated following the same procedure. After removal of the second round PDMS microfluidic chip, slides were washed with DEPC water, dried and aligned on the tissue with a round perforated PDMS piece. A lysis solution (50 μL of 1 × PBS, 50 μL of 2 × lysis buffer (20 mM Tris pH 8.0, 400 mM NaCl, 100 mM EDTA, 4.4% SDS, NFH_2_O), and 10 μL of proteinase K solution (20 mg/mL)) was added, and lysis was carried out at 55 °C for 2 hours. Subsequently, the lysate was collected and stored at -80 °C for later use.

The cDNAs were purified in the lysate using DB MyOne streptavidin C1 beads (Invitrogen, 65002). The beads were first washed three times with 1 × B&W buffer (0.5 μL of Tris pH 8.0, 20 μL of 5 M NaCl, 0.1 μL of 0.5 M EDTA, and 79.4 μL of ddH_2_O). To perform purification from stored tissue lysate, an equal volume of water was added to reduce the SDS concentration. Purification was carried out using the DNA Clean and Concentrator kit (Zymo, D4014). Next, 100 μL of the cleaned MyOne C1 bead suspension was added to the purification sample and incubated at room temperature for 60 min with gentle rotation. The beads with cDNA were further cleaned twice with 1 × B&W buffer containing 0.05% Tween-20 and SUPERase In RNase Inhibitor for 5 min each, followed by one wash with STE buffer (500 μL of Tris pH 8.0, 500 μL of 5 M NaCl, 100 μL of 0.5 M EDTA, and 48.9 μL of ddH_2_O) for 5 min with rotation. The beads with cDNA were then ready for template switching.

The cDNAs bound to beads were purified and resuspended in the template switch solution. The template switch reaction mix consisted of 93.5 μL of 30% PEG6000, 44 μL of Maxima RT buffer (Thermo Fisher, EP0753), 44 μL of Ficoll PM-400 solution (Sigma-Aldrich, F5415), 11 μL of dNTPs (Thermo Fisher, N0447S), 5.5 μL of RNase Inhibitor (Enzymatics, Y9420L), 11 μL of Maxima H Minus Reverse Transcriptase (Thermo Fisher, EP0753), and 11 μL of template switch primer. The reaction was carried out at room temperature for 30 minutes followed by incubation at 42 °C for 90 minutes. After template switching, beads were washed once with STE buffer and then once more with 400 μL of RNase-free water. The supernatant was removed by placing the sample on a magnetic stand. Beads with cDNA were then amplified by resuspending them in a PCR mix containing 1× Kapa HiFi PCR mix (KAPA Biosystems, KK2601), 400 nM of P7 primer, and 400 nM of RNA PCR primer. The PCR reaction was carried out under the following conditions: 95 °C for 3 minutes, followed by 6-10 cycles of 98 °C for 30 seconds, 65 °C for 45 seconds, and 72 °C for 3 minutes.

After amplification, the sample was centrifuged at 10,000 g for 1 minute, and the supernatant was transferred to a new tube. It was then purified using 0.8 × SPRI select beads (Beckman Coulter, B23318) and eluted to 36 μL of RNase-free water as per the manufacturer's instructions. The amount of cDNA was quantified using a Qubit fluorometer (Thermo Fisher, Q33238). For each sample, 50 ng of cDNA was fragmented in a 50 μL tagmentation mix at 55 °C for 10 minutes. An equal volume of 8 M Gu·HCl (Solarbio, G8070) was added, and the cDNA was purified using 2 × SPRI beads and eluted in RNase-free water. Subsequently, the purified cDNA was mixed with a tagmentation PCR mix consisting of 25 μL of NEB Next High-Fidelity 2 × PCR Master Mix, 2.5 μL of 10 μM P7 primer, and 2.5 μL of 10 μM Ad1 primer with sample-specific barcodes. PCR was performed under the following conditions: 72 °C for 5 minutes, 98 °C for 30 seconds, and then 7-9 cycles of 98 °C for 10 seconds, 65 °C for 30 seconds, and 72 °C for 1 minute. The amplified library was purified using 0.7 × SPRI select beads and eluted in 12 μL of RNase-free water. Libraries were quantified using a Bioanalyzer (Agilent) and Qubit fluorometer (Thermo Fisher), and then sequenced on a NovaSeq 6000 (Illumina, San Diego, CA) with 150-bp paired-end reads.

UMI-tools v1.1.0 was used to extract barcode A, barcode B, and UMI from read2 for generating a spatial gene expression matrix for tissue 35. Subsequently, the reshuffled fastq files were processed using ST pipeline v1.8.1 36, 37, which included adapter trimming, read alignment to the reference genome (GRCm38), annotation (Ensembl release 102), and UMI and gene counting. Seurat V4 was employed to normalize 38, reduce dimensions, cluster, and annotate the spatial expression matrix of genes. A spatial expression map of the tissue was then generated based on barcodes. Mesenchymal cells of ovulating follicles at stages two to four and non-ovulatory area groups were grouped, respectively. The ring of mesenchymal cells was defined to be located within 40 μm from the ovulatory point as periovulatory mesenchymal cells (black box of Figure 3E), while the remaining mesenchymal cells were categorized as non-ovulatory area mesenchymal cells. Differential expression genes between matrix cells near the ovulatory point and those distant from it were subjected to GO and KEGG analyses using cluster Profiler 39. Additional details on spatial transcriptome sequencing can be found in Table S1.

### 2.7 Whole ovary culture

7 dpp mouse ovaries were utilized to conduct the assay. The ovaries of mice were injected with rCTSL protein (Abcam, ab198444) using a capillary glass needle. Subsequently, the injected ovaries were cultured in 6-well plates, supplemented with Dulbecco Modified Eagle Medium/Ham F12 nutrient mixture (DMEM/F12) (Gibco, Life Technologies, CA). The culture conditions were maintained at 37 °C, 5% CO_2_, and saturated humidity for a period of 2 days.

For the incubation of whole ovaries with Z-FY-CHO, the 7 dpp ovaries of mice were treated with 10 μM Z-FY-CHO (MCE, HY-128140) and cultured under similar conditions for 2 days at 37 °C, 5% CO^2^, and saturated humidity.

Procedure of incubation of whole ovary with DMSO 48 h + CTK 1 hour: The 7 dpp ovaries of mice were cultured in DMEM/F12 medium containing DMSO for 2 days and then treated with CTK diluted by DMEM/F12 for 1 hour at 37°C, 5% CO2 and saturated humidity. CTK reagent was prepared as (1 μM CaCl2, collagenase type IV (0.1 mg/mL), 20% KSR (Invitrogen), and 0.025% trypsin EDTA (Invitrogen).

Procedure of incubation of whole ovary with Z-FY-CHO 48 h + CTK 1 hour: The 7 dpp ovaries of mice were treated with 10 μM Z-FY-CHO (MCE, HY-128140) for 2 days at 37°C, 5% CO2 and saturated humidity, then treated with CTK for 1 h at 37°C, 5% CO2 and saturated humidity.

### 2.8 Ovarian topical administration *in vivo*

According to Zhang's protocol 15, female mice were anesthetized with avertin (300 mg/kg, T48402, Sigma, USA) prior to surgery. In the control group, we injected precooled growth factor-reduced Matrigel (354230, BD, USA) into the unilateral ovarian bursa using an insulin syringe. The contralateral ovary was injected with Matrigel containing either rCTSL or Z-FY-CHO, as appropriate. Once the temperature sensitive Matrigel had solidified, the incisions were sutured.

### 2.9 Oocyte nucleus rotation

According to Nagamatsu's protocol 26, Ovaries were stained with Hoechst 33342 (BIYUNTIAN) at 37 °C for 30 minutes in a CO_2_ incubator set to 5% CO_2_. Subsequently, they were transferred to dishes containing Prolong antifade solution (Invitrogen). Time-lapse imaging was then conducted using a Nikon A1 microscope, with images captured every 20 seconds for up to 30 minutes, resulting in a maximum of 90 images.

### 2.10 Statistical analysis

All experiments were biologically replicated at least three times. The results are presented as the mean ± SD and were analyzed using GraphPad Prism 8. The data was analyzed using a *t*-test and considered statistical significance at p < 0.05.

## 3. Results

### 3.1 PmFs closer to ovulatory follicles are activated before more distant PmFs in mice *in vivo*

**Figure 1:**
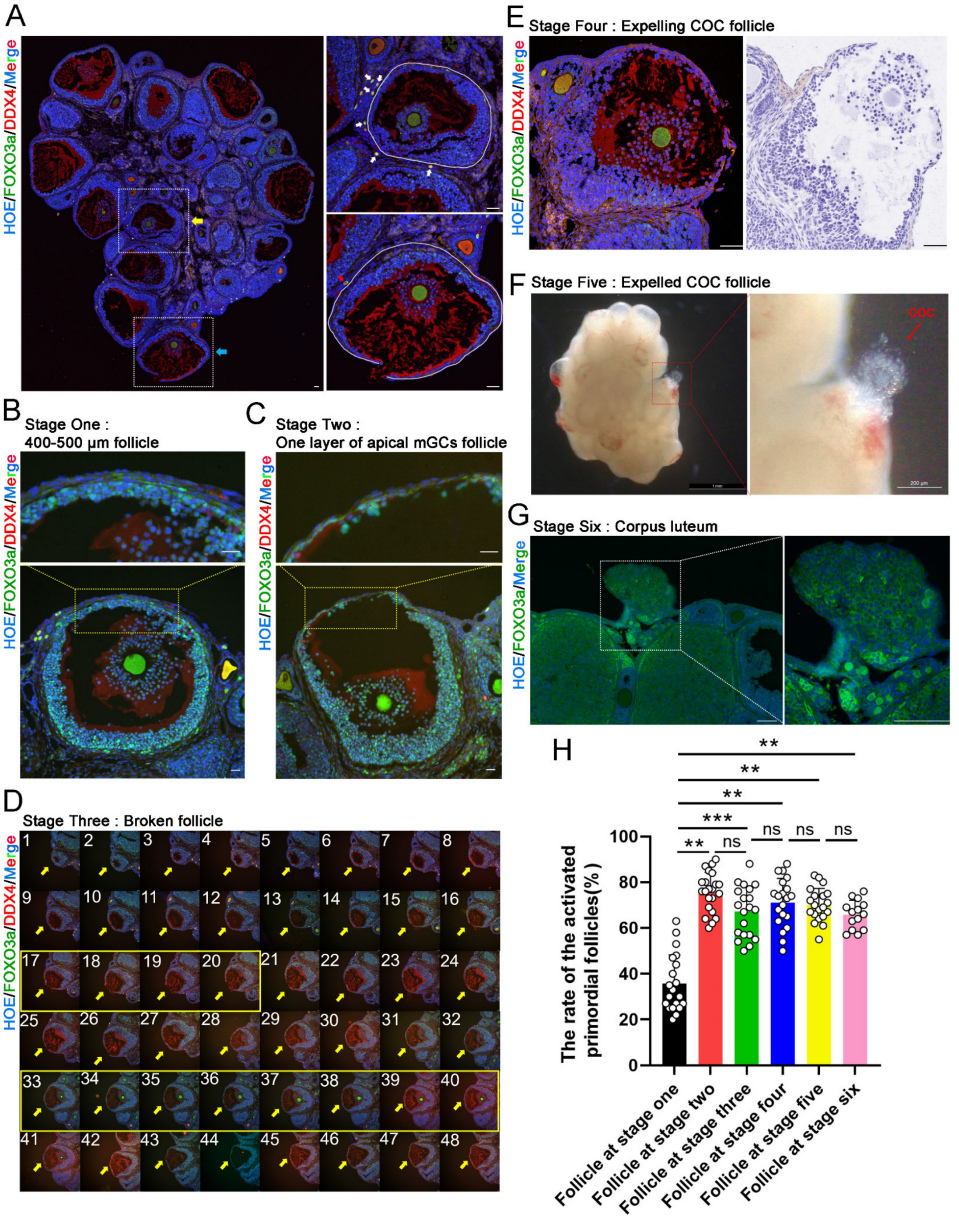
** PmFs closer to ovulatory follicles are activated before more distant PmFs in mice *in vivo*. (A)** Histological outlines of adult mice ovaries. The yellow arrow indicates a growing antral follicle and the blue arrow indicates a ruptured ovulatory follicle. Solid white lines describe the margins of follicles. The oocyte cytoplasm is indicated by DDX4 (red) and the nucleus of each cell is dyed with Hoechst (blue). PmFs with co-localized FOXO3a (green) and DDX4 in the oocyte cytoplasm are activated PmFs, while PmFs with nucleic localized FOXO3a are PmFs in a non-growing state. Scale bar = 40 μm. **(B)** Stage one ovulatory follicle with a diameter range of 400-500 μm. 3 or 4 layers of apical mGCs remained in the follicular wall. Scale bar = 40 μm. **(C)** Stage two ovulatory follicle. Scale bar = 40 μm. **(D)** Stage three ovulatory follicle. Pictures 1-48 are continuous tissue sections of the broken follicle. Pictures 17-20 show the ruptured structure of the follicle. Pictures 33-40 indicate the location of the COC, which remained inside the follicle. Scale bar = 40 μm. **(E)** Stage four ovulatory follicle with expelling COC. Scale bar = 100 μm. **(F)** Stage five ovulatory follicle whose COC was just expelled out. **(G)** Stage six ovulatory follicle. The dashed white box indicates the corpus luteum. **(H)** Activation ratio of PmFs within a 40 μm range adjacent to the differently stages follicles based on follicular counting. n = 14. FOXO3a (green), DDX4 (red), Hoechst (blue). Results are presented as mean ± SEM and analyzed by a Student's *t*- test, two-sample unpaired. *p < 0.05; **p < 0.01; ns ≥ 0.05, no significant difference.

To determine whether selective activation of PmFs under physiological conditions is correlated with cyclic ovulation events in mice, we classified the LH-induced ovulation process into six consecutive stages. Briefly, at stage one, ovulatory follicles were 400-500 μm in diameter, with a complete follicular structure, and with cumulus cells surrounding the oocyte beginning to expand (Figure 1B); stage two ovulatory follicles were defined as those forming one-cell-layer walls comprised of apical mural granulosa cells (mGCs) (Figure 1C); stage three ovulatory follicles were those with ruptured follicular walls (Figure 1D); stage four ovulatory follicles were defined as those with ruptured follicular walls and a cumulus oocyte complex (COC) migrating to the rupture in the wall (Figure 1E); stage five ovulatory follicles were those with COC expelled to the exterior ovarian surface (Figure 1F); and stage six ovulatory follicles were those follicles with a cavity filled by corpus luteum cells (Figure 1G).

Based on this classification, we compared the proportion of PmFs near ovulatory follicles that were activated with the proportion of activated PmFs near growing follicles. Given the ~20 μm diameter of PmFs, 40 μm was sufficient to cover two full-sized PmFs on a tissue slide. Therefore, we used 40 μm from the edge of the basal membrane of ovulatory follicles or growing follicles as the cut-off for identifying activated PmFs and to rule out the PmFs been activated by neighboring follicles located more than 40 μm to the PmFs (Figure 1A). We found that the percentage of activated PmFs was significantly higher in stages two through six than that of stage one ovulatory follicles (35% versus 75%, 67%, 71%, 70%, and 66%; stage 1 versus 2, 3, 4, 5, 6; p < 0.01; Figure 1H), while the proportions of activated PmFs did not significantly differ among stages 2-6. These results suggested that PmFs may be selectively activated in a manner dependent on events in ovulation, with those PmFs closer to ovulatory follicles activated earlier than more distant PmFs in mouse ovaries *in vivo*.

### 3.2 Both consecutive superovulation (CSO) and consecutive restrained ovulation (CRO) affected PmF reserve

**Figure 2:**
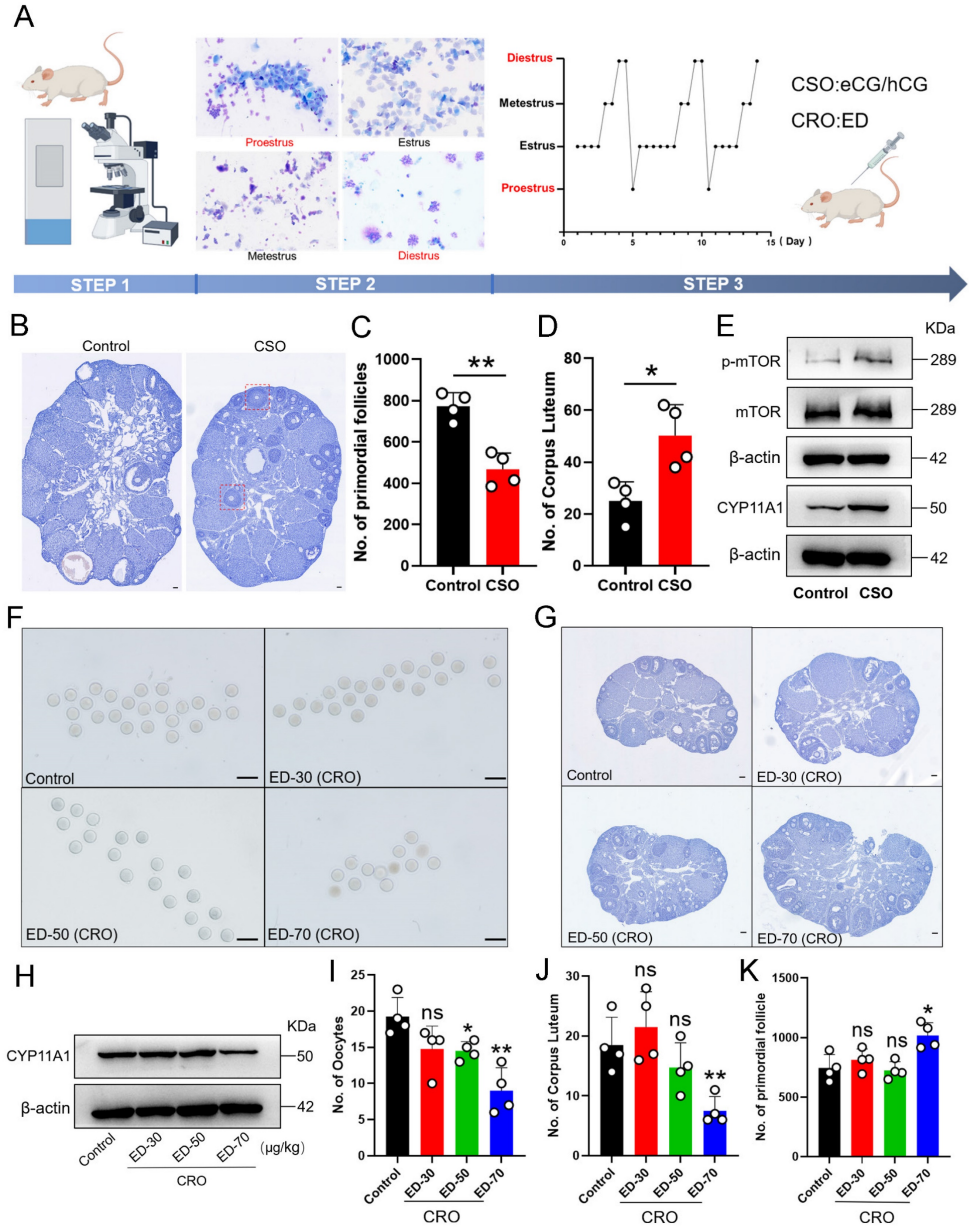
** Both consecutive superovulation (CSO) and consecutive restrained ovulation (CRO) affected PmF reserve. (A)** *In vivo* intervention procedures of female estrous cycles. CSO was performed through eCG/hCG stimulation. CRO was performed through intraperitoneal ED injection. Before treatments, the estrous cycle of 6-8 weeks old ICR females were identified via a vaginal smear. Mice at proestrus or diestrus stages were used in the following assays. Picture was drawn on Figdraw. **(B)** Hematoxylin staining of the ovaries. Red boxes indicate abnormal follicles found in the CSO group. Scale bar = 40 μm. **(C)** Number of PmF after CSO treatment. **(D)** Number of corpus luteum after CSO treatment. **(E)** Expression levels of p-mTOR, mTOR, and CYP11A1 proteins after CSO treatment. **(F)** Oocytes from the oviduct of mice after CRO treatment. **(G)** Overview of mice ovaries treated with ED of different concentrations. **(H)** Protein levels of CYP11A1 after CRO treatment. **(I)** Ovulation data analysis of mice treated with ED in **(F)**. **(J)** Number of corpus luteum after ED treatments. **(K)** Number of PmFs after ED treatments. All experiments were biologically replicated more than 3 times. Results are presented as mean ± SEM and analyzed by a Student's *t*- test, two-sample unpaired. *p < 0.05; **p < 0.01; ns p ≥ 0.05.

To next investigate whether consumption of the PmF reserve in mouse ovaries was related to the number of ovulations in reproductive age female mice, we induced consecutive superovulation (CSO) for 10 consecutive estrus cycles in mice. To this end, we treated 6-8-week-old female ICR mice at either proestrus or diestrus with eCG plus hCG to induce CSO with corresponding treated with physiological saline mice serving as controls (Figure 2A). Then, total PmFs were counted in consecutive sections of whole ovaries for statistical analysis. Following CSO, we observed that residual corpus luteum were significantly more abundant compared to that in ovaries of control mice, while fewer available PmFs could be detected in CSO ovaries relative to controls (Figure 2B-2D), implying that superovulation accelerated the depletion of PmFs. Further, CSO ovaries had a higher number of abnormal follicles compared to control ovaries, and these abnormal follicles formed multiple layers of GCs but failed to develop into antral follicles (Figure 2B).

In agreement with these findings, Western blots indicated that protein levels of the corpus luteum functional marker, CYP11A1, were significantly elevated in response to gonadotropin stimulation after CSO treatment (Figure 2E). These results confirmed that superovulation treatment indeed resulted in more ovulation and promoted formation of functional corpus luteum. In addition, the levels of the activated PmF marker, phosphorylated mTOR (p-mTOR), were also significantly elevated in CSO-treated mice (Figure 2E). Collectively, these results supported that superovulation treatment could accelerate the rate of depletion of PmF reserves in mouse ovaries.

Based on the effects we observed in CSO-treated mice, we next induced consecutive restrained ovulation (CRO) in 6-8-week-old female ICR mice at either proestrus or diestrus through treatment with ethynodiol diacetate (ED). In contrast with CSO assays, oocyte counts revealed that ED-treated mice (50, 70 μg/kg) had significantly fewer mature oocytes than control animals (Figure 2F, 2I), while significantly fewer corpus luteum could be detected (Figure 2J). Similarly, Western blots showed that CYP11A1 protein levels were also significantly decreased in CRO group ovaries (ED-70 μg/kg) relative to controls (Figure 2H). The CRO mice (ED-70 μg/kg) also had significantly higher numbers of PmFs compared to controls (Figure 2G, 2K), which aligned well with the decrease in mature oocytes. Collectively, these results suggested that CRO treatment could delay activation of PmFs, which ultimately decreased the depletion rate of PmF reserves.

### 3.3 Ovulation involves significant degradation and remodeling of ECM of mesenchymal cells within 40 μm of the follicle

**Figure 3:**
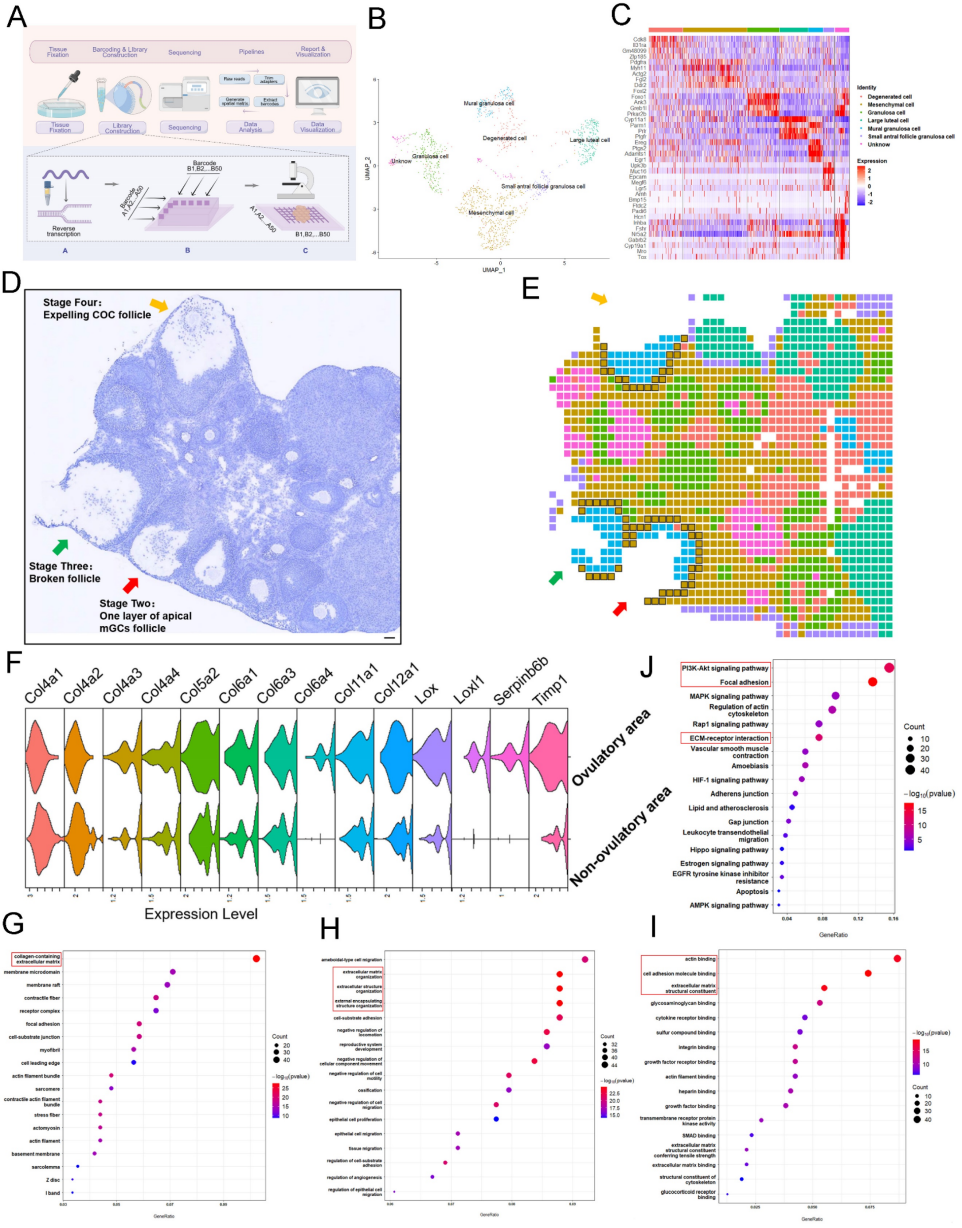
** Ovulation involves significant degradation and remodeling of ECM of mesenchymal cells within 40 μm of the follicle. (A)** Spatial transcriptome profiling workflow depicting the sample collection, tissue handling, library preparation, sequencing, and analysis of ovarian tissues collected from mice 12 hours after hCG priming. Picture is drawn on Figdraw. **(B)** UMAP clustering showing that the ovarian cells belong to different clusters. **(C)** Highly expressed genes specific to each cluster.** (D, E)** Overview of the ovarian slides selected for examining of the spatial transcriptomics of the ovulating ovary. **(D)** Hematoxylin staining of the selected section of ovarian tissue. Arrows indicate ovulating follicles. Scale bar = 40 μm. **(E)** Cellular spatial transcriptome map corresponding to **(D)**. Different colors represent various cell clusters in the same slide. **(F)** mRNA levels of ECM-related genes in ovulatory and non-ovulatory regions. **(G-I)** Gene ontology (GO) analysis showing the most significantly altered transcripts of either cellular component **(G)**, biological process **(H)**, or molecular function **(I)**, within the 40 μm range of mesenchymal cells adjacent to the ovulating follicle in comparison to those found in mesenchymal cells outside the 40 μm range. ECM-related genes were the mostly enriched cells. **(J)** KEGG analysis of mesenchymal cells close to the ovulatory or non-ovulatory area. All experiments were biologically replicated more than 3 times. Results are presented as mean ± SEM and analyzed by a Student's *t*- test, two-sample unpaired. *p < 0.05; **p < 0.01; ns p ≥ 0.05.

To better understand the molecular basis leading to selective activation of closer PmFs, we conducted single-cell spatial transcriptome sequencing of the area around ovulating follicles and those outside these areas of 3-week-old mice by modifying an established DBit-seq approach (Figure 3A) 37.

In brief, mouse ovaries treated with eCG, then 46 hours later were administered hCG and ovaries were collected 12 hours later to prepare 10 μm cryosections. Principal component analysis (PCA; Figure 3B) with hierarchical clustering and heatmap visualization (Figure 3C) identified seven cell types based on signature marker gene expression. The spatial organization of these cell types matched well with anatomical structures observed in HE staining images (Figure 3D, 3E), with mesenchymal cells, (defined by *Pdgfrα* and *Myh11* expression) distributed around the follicles and populating the ovarian stromal region (Figure 3E).

To identify differentially expressed genes (DEGs) between tissues adjacent to ovulating follicles (within 40 μm) and other ovarian regions (Table S1), we manually classified mesenchymal cells as ovulatory area group (120 total) or non-ovulatory area group (714 total) according to their distance from ovulating follicles (Figure 3E). GO functional annotations showed enrichment in terms associated with ECM degradation and remodeling such as wound healing, cell substrate adhesion, growth factor binding and heparin binding among upregulated DEGs in mesenchymal cells adjacent to ovulating follicles (Figure 3G-3I). KEGG analysis further illustrated that the most significant pathways in ovulatory area were enriched with DEGs involved in actin binding, degradation, and remodeling in ECM-related events (e.g., *Col12a1, Lox, Timp1*) compared to their expression in non-ovulatory area (Figure 3F, 3J, Table S2). These genes have been shown to participate in reshaping or regulating ECM structure 40-42. Collectively, these results suggested that the ovulation process entailed ECM degradation and remodeling around mesenchymal cells within 40 μm of ovulating follicles.

### 3.4 Ovulation related ECM changes correlated to the activation of the PmFs within the 40 μm range of adjacent ovulating follicle

**Figure 4:**
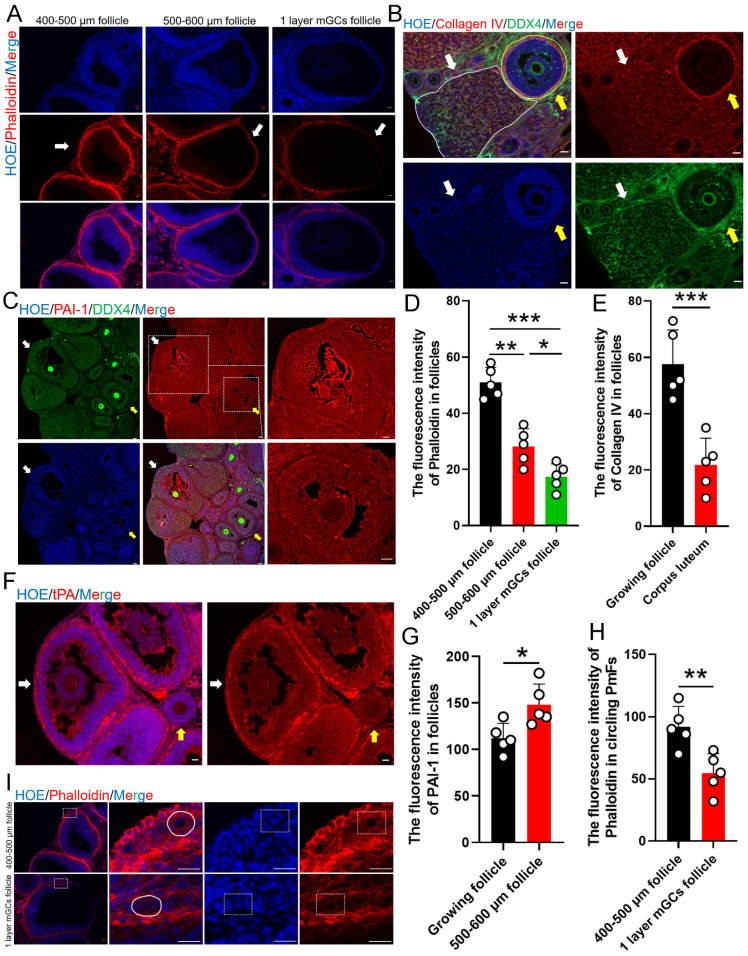
** Ovulation related ECM changes correlated to the activation of the PmFs within the 40 μm range of adjacent ovulating follicle.** Changes to mechanical stress of the surrounding follicles are indicated by changes in fluorescence intensity of phalloidin. **(A)** Phalloidin fluorescently stained follicles with diameters of 400-500 μm antral follicles, 500-600 μm antral follicles, and stage two ovulatory follicles. Phalloidin (red), Hoechst (blue). Scale bar = 20 μm. **(B)** Immunofluorescent staining of Collagen IV. White arrows and solid line indicate the corpus luteum. Yellow arrows and solid lines indicate growing antral follicles. Collagen IV (red), DDX4 (green), Hoechst (blue). Scale bar = 40 μm. **(C)** Immunofluorescent staining of PAI-1. The yellow arrows indicate growing antral follicles, and the white arrows indicate 500-600 μm antral follicles. PAI-1 (red), DDX4 (green), Hoechst (blue). Scale bar = 40 μm. **(D)** Fluorescence intensity of follicle Phalloidin in (A) was analyzed by Image J software. **(E)** Fluorescence intensity of follicle Collagen IV in **(B)**. **(F)** Immunofluorescent staining of tPA. tPA (red), Hoechst (blue), Scale bar = 20 μm. **(G)** Fluorescence intensity of PAI-1 in **(C)**. **(H)** Fluorescence intensity of Phalloidin in **(I)**. **(I)** Phalloidin fluorescently stained the PmFs around antral follicles of 400-500 μm in diameters and stage two ovulatory follicles. White solid line and dashed box both indicates the PmFs. Phalloidin (red), Hoechst (blue). Scale bar = 20 μm. All experiments were biologically replicated more than 3 times. Results are presented as mean ± SEM and analyzed by a Student's *t*-test, two-sample unpaired. The fluorescence intensities of target proteins were analyzed by the Image J software. *p < 0.05; **p < 0.01; ns p ≥ 0.05.

Given that the most significant transcriptomic changes associated with ovulation involved terms related to ECM, which is known to play a key role in the formation of contractile stress fibers that increase tension, especially in tissue around PmFs that contributes to the non-growing state, indicated by the observed rotation of the oocyte nucleus 26. To therefore explore whether the changes in mechanical stress around ovulating follicles could participate in activating nearby PmFs, we first examined mechanical stress in ovaries at estrus using fluorescence-labeled phalloidin to stain F-actin 43. This analysis showed that phalloidin intensity, and therefore mechanical stress, was significantly lower around stage two follicles compared to large antral follicles (diameter of 400-600 μm; Figure 4A, 4D). At the same time, immunofluorescent signal intensity for collagen IV, a primary component of the ECM, was significantly weaker around corpus luteum than that around non-ovulating follicle (Figure 4B, 4E). In addition, the plasminogen, tPA, which hydrolyzes protein in conjunction with its inhibitor, PAI-1, were both expressed at markedly higher levels near ovulating follicles compared to non-ovulating follicles (Figure 4C, 4F, 4G).

Assessment of mechanical stress, based on phalloidin staining, PmFs adjacent to stage two ovulating follicles showed significantly lower signal intensity than the PmFs adjacent to 400-500 μm diameter antral follicles (Figure 4H, 4I). Finally, examination of nuclear rotation rates of PmFs during ovulation in eCG/hCG-treated ovaries showed that it was significantly slower than that of PmFs in non-hormonally treated ovaries (Video1-2, Figure S1A-C). This finding was in agreement with a previous study that reported faster nuclear rotation rates are associated with lower likelihood of PmF activation 26.

These findings suggested that proteases accumulate to higher levels around ovulating follicles than non-ovulating follicles, potentially contributing to ECM degradation, which could locally relax tension around nearby PmFs and lead to activation of PmFs.

### 3.5 PmFs closest to ovulating follicles show greater enrichment with CTSL protein, which can activate PmFs

**Figure 5:**
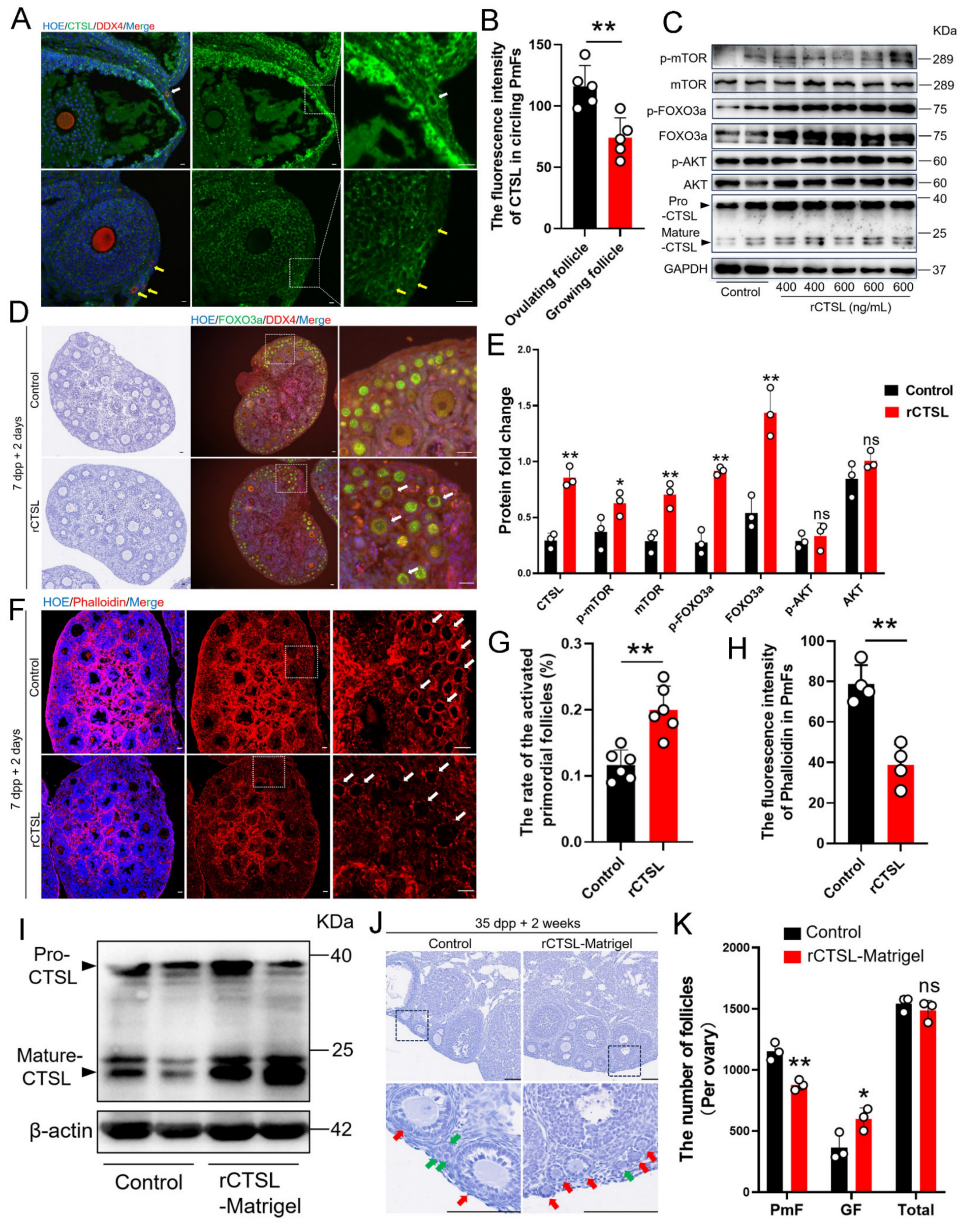
** PmFs closest to ovulating follicles show greater enrichment with CTSL protein, which can activate PmFs. (A)** Immunofluorescent staining of CTSL. The white arrows indicate the PmFs around the ovulating follicle, and the yellow arrows indicate the PmFs around the non-ovulating follicle (Growing follicle). DDX4 (red), CTSL (green), Hoechst (blue). Scale bar = 20 μm. **(B)** Fluorescence intensity of CTSL circling the PmFs. **(C)** Western blot analysis of the effect of intra ovarian injection of rCTSL protein on the levels of p-mTOR, mTOR, p-FOXO3a, FOXO3a, pro-CTSL, mature-CTSL, p-AKT, and AKT proteins. **(D)** Hematoxylin staining and immunofluorescence staining of ovaries of 7 dpp mice injected with rCTSL and cultured for 2 days. White arrows indicate activated PmFs. DDX4 (red), FOXO3a (green), Hoechst (blue). Scale bar = 20 μm. **(E)** Quantification of ratio respective proteins normalized to GAPDH in **(C)**. **(F)** Immunofluorescent staining of Phalloidin. White arrows indicate cortical PmFs. Phalloidin (red), Hoechst (blue). Scale bar = 20 μm. **(G)** Activation ratio of PmFs based on follicular counting based on **(D)**. **(H)** Fluorescence intensity of Phalloidin in cortical PmFs based on **(F)**. **(I)** Western blot analysis showing that the level of mature-CTSL protein was significantly increased by *in situ* injection of rCTSL-Matrigel into the ovarian bursa. **(J)** Hematoxylin staining of ovaries of 35 dpp mice injected with rCTSL and cultured for 2 weeks. Red arrows indicate growing follicles. Green arrows indicate PmFs of non-growing state. Scale bar = 100 μm. **(K)** Number of follicles in rCTSL-Matrigel groups and Control groups. GF, Growing Follicle. Total, All Follicles. All experiments were biologically replicated more than 3 times. Results are presented as mean ± SEM and analyzed by a Student's *t*-test, two-sample unpaired. The fluorescence intensities of target proteins were analyzed by the Image J software. *p < 0.05; **p < 0.01; ns ≥ 0.05.

After screened the changed ECM related genes derived from our spatial transcriptome results, the analysis showed that *Cathepsin L* (*Ctsl*) was also significantly upregulated in the 40 μm area around ovulating follicles compared to its expression in more distant, non-ovulating regions (Figure S2A). Immunofluorescence staining showed that CTSL protein was indeed highly expressed in mGCs of ovulating follicles (Figure S2B, S2C), and was enriched to higher levels in PmFs adjacent to ovulating follicles than in PmFs near non-ovulating follicles (Figure 5A, 5B). These findings led us to hypothesize that CTSL might play a key role in degrading ECM around PmFs adjacent to ovulating follicles *in vivo*.

To explore this possibility, we collected ovaries of neonatal mice at 7 days post-partum (dpp) to test whether exposure to higher CTSL levels affected the rate of PmF activation. For this purpose, 7 dpp ovaries were injected with purified recombinant CTSL (rCTSL) protein and incubated for 2 days. Histological analysis of PmF activation, based on the FOXO3a marker, showed that phosphorylated FOXO3a (p-FOXO3a) primarily localized to the oocyte cytoplasm of PmFs, whereas un-phosphorylated FOXO3a generally localized in nuclei, which aligned with FOXO3a inhibitory function in preventing premature oocyte activation 16-18. The significantly higher percentage of FOXO3a cytoplasmic localized follicles in sections of rCTSL-treated whole ovaries compared to that in control ovaries suggested that exposure to rCTSL could stimulate PmF activation (Figure 5D, 5G). Further evaluation by Western blotting indicated that ovarian protein levels of PmF activation markers (i.e., mTOR, p-mTOR, FOXO3a and p-FOXO3a) were significantly increased in the rCTSL treatment group compared to Controls (Figure 5C, 5E). Ultimately, activated PmFs were significantly more abundant in whole ovaries injected with rCTSL compared to Controls. Phalloidin staining also showed lower signal intensity in circling the PmFs in the ovarian cortex of rCTSL-treated ovaries than that around PmFs in Control ovaries (Figure 5F, 5H). These results suggested that CTSL mediates critical functions leading to the activation of PmFs adjacent to ovulating follicles in mouse ovaries.

Given these effects of rCTSL in 7 dpp mouse ovaries, we next investigated whether injecting a rCTSL-containing liquid Matrigel into the ovarian bursa of 35 dpp mice could similarly stimulate prolonged PmF activation in mouse ovaries* in vivo* (Figure S2D). Two weeks after the surgery, Western blots confirmed that ovarian protein levels of Mature-CTSL were significantly higher in the rCTSL-Matrigel group compared to controls (Figure 5I). More growing follicles were observed in the ovarian cortex of rCTSL-Matrigel mice, while the Matrigel-only control ovaries had more non-growing follicles (PmFs) compared to the rCTSL-Matrigel group (Figure 5J-K). These results suggested that CTSL could promote PmF activation in ovaries of reproductive age mice *in vivo*.

### 3.6 The CTSL induced activation of PmFs that are adjacent to ovulating follicles via ECM degradation is one of possible mechanisms responsible for PmF activation *in vivo*

**Figure 6:**
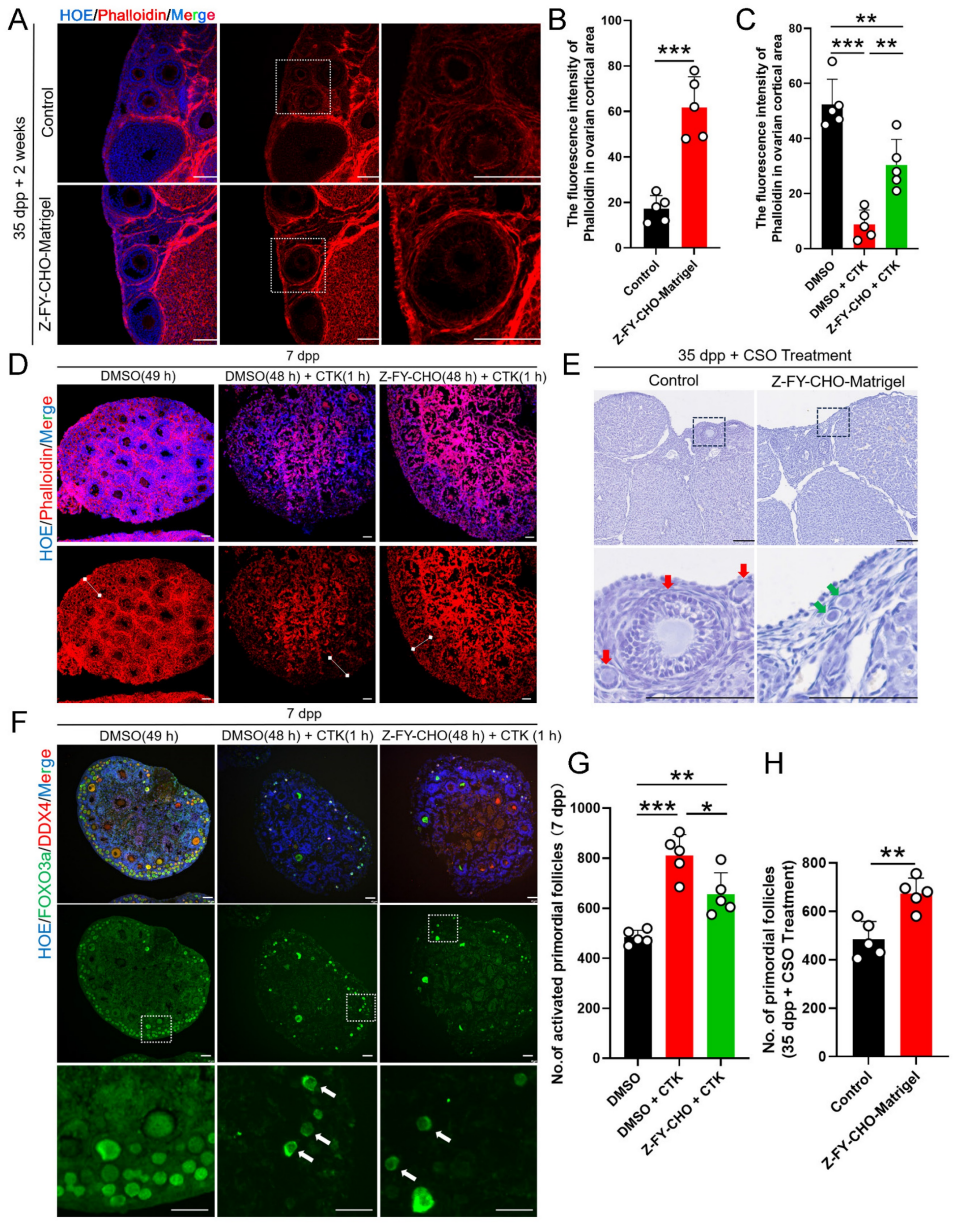
** The CTSL induced activation of PmFs that are adjacent to ovulating follicles via ECM degradation is one of possible mechanisms responsible for PmF activation *in vivo*. (A)** Immunofluorescent staining of Phalloidin in Control groups and Z-FY-CHO-Matrigel groups. White boxes indicate ovarian cortical. Phalloidin (red), Hoechst (blue). Scale bar = 100 μm. **(B)** Fluorescence intensity of Phalloidin in cortical based on (A). **(C)** Fluorescence intensity of Phalloidin in the ovarian cortex of Control groups, CTK-treated groups, and Z-FY-CHO+CTK-treated groups based on **(D)**. **(D)** Immunofluorescent staining of Phalloidin in Control groups (DMSO 49 hours), CTK-treated groups (DMSO 48h + CTK 1h), and Z-FY-CHO+CTK-treated groups. White lines indicate ovarian cortical areas. Phalloidin (red), Hoechst (blue). Scale bar = 40 μm. **(E)** Hematoxylin staining of ovaries of 35 dpp mice injected with Z-FY-CHO-Matrigel and processed with CSO. Red arrows indicate growing follicles. Green arrows indicate PmFs. Scale bar = 100 μm. **(F)** Immunofluorescent staining of Control, CTK-treated, and Z-FY-CHO+CTK-treated groups. White arrows indicate activated PmFs in the ovarian cortex areas. DDX4 (red), FOXO3a (green), Hoechst (blue). Scale bar = 40 μm. **(G)** Number of activated PmFs (primary follicle). **(H)** Number of PmFs in Z-FY-CHO-Matrigel groups and Control groups after CSO treatment. All experiments were biologically replicated more than 3 times. Results are presented as mean ± SEM and analyzed by a Student's *t*-test, two-sample unpaired. The fluorescence intensities of target proteins were analyzed by the Image J software. *p < 0.05; **p < 0.01; ns ≥ 0.05.

Given the above evidence of CTSL function in activating PmFs in the immediate vicinity of ovulating follicles, we next sought to determine whether CTSL was indispensable for PmF activation by treating 7 dpp mouse ovaries with Z-FY-CHO, a specific inhibitor of CTSL, for two days* in vitro*. Phalloidin staining in Z-FY-CHO-treated ovaries indicated that inhibition of CTSL led to significantly denser F-actin signal in the ovarian cortex surrounding PmFs compared with that in Control (Figure S3A, S3B). Furthermore, after inhibition of CTSL, the protein levels of p-FOXO3a in the ovary decreased significantly as well (Figure S3C, S3D). Similarly, at 2 weeks following treatment of 35 dpp mice with Z-FY-CHO-Matrigel, phalloidin staining in ovarian sections showed that the F-actin network was significantly denser in the ovarian cortex around PmFs compared with Matrigel-only controls (Figure 6A, 6B). These results implied that inhibition of CTSL have strengthened the ECM around the PmF.

To further test the effects of CTSL in PmF activation, we next administered 7 dpp ovaries with a treatment for digesting ovarian ECM *in vitro*. CTK is a solution that can specifically digest ovarian ECM, comprising collagenase type IV, trypsin, and knockout serum replacement (KSR), was induce PmF activation in previous reports 26. F-actin staining in 7 dpp mouse ovaries treated with DMSO 49 h, or DMSO 48 h + CTK 1 hour, or Z-FY-CHO 48 h + CTK 1 hour respectively showed that CTK treatment resulted in almost abolishing the F-actin network in the ovarian cortex compared to the Control (DMSO 49 h) (Figure 6C, 6D), whereas ovaries exposed to Z-FY-CHO for 48 h prior to CTK had obviously higher signal from phalloidin staining (Figure 6C, 6D), indicating that ECM damage by CTK treatment was attenuated by the CTSL inhibitor. In addition, Western blots showed that p-FOXO3a levels were significantly higher in the DMSO+CTK group (Figure S3E, S3F), while immunofluorescence staining confirmed that FOXO3a primarily localized in the cytoplasm of oocytes in DMSO+CTK ovaries compared to control ovaries (DMSO) (Figure 6F, 6G), supporting that F-actin degradation around PmFs led to their activation. In addition, PmF counts also showed that ovaries treated with Z-FY-CHO+CTK had fewer activated PmFs compared to the DMSO+CTK group (Figure 6G). These results suggested that ECM degradation by CTSL or CTK treatment could enhance PmF activation, while inhibiting ECM degradation by Z-FY-CHO could reduce PmF activation in *vitro* mouse ovaries. In 35 dpp mouse ovaries injected with Z-FY-CHO-Matrigel *in vivo* followed by CSO treatment for 10 estrus cycles, the number of non-growing PmFs in the ovaries treated with Z-FY-CHO-Matrigel was significantly higher than the Matrigel-only control ovaries, indicating that inhibiting CTSL could effectively attenuate PmF overactivation arising from superovulation treatment (Figure 6E, 6H).

## 4. Discussion

**Figure 7:**
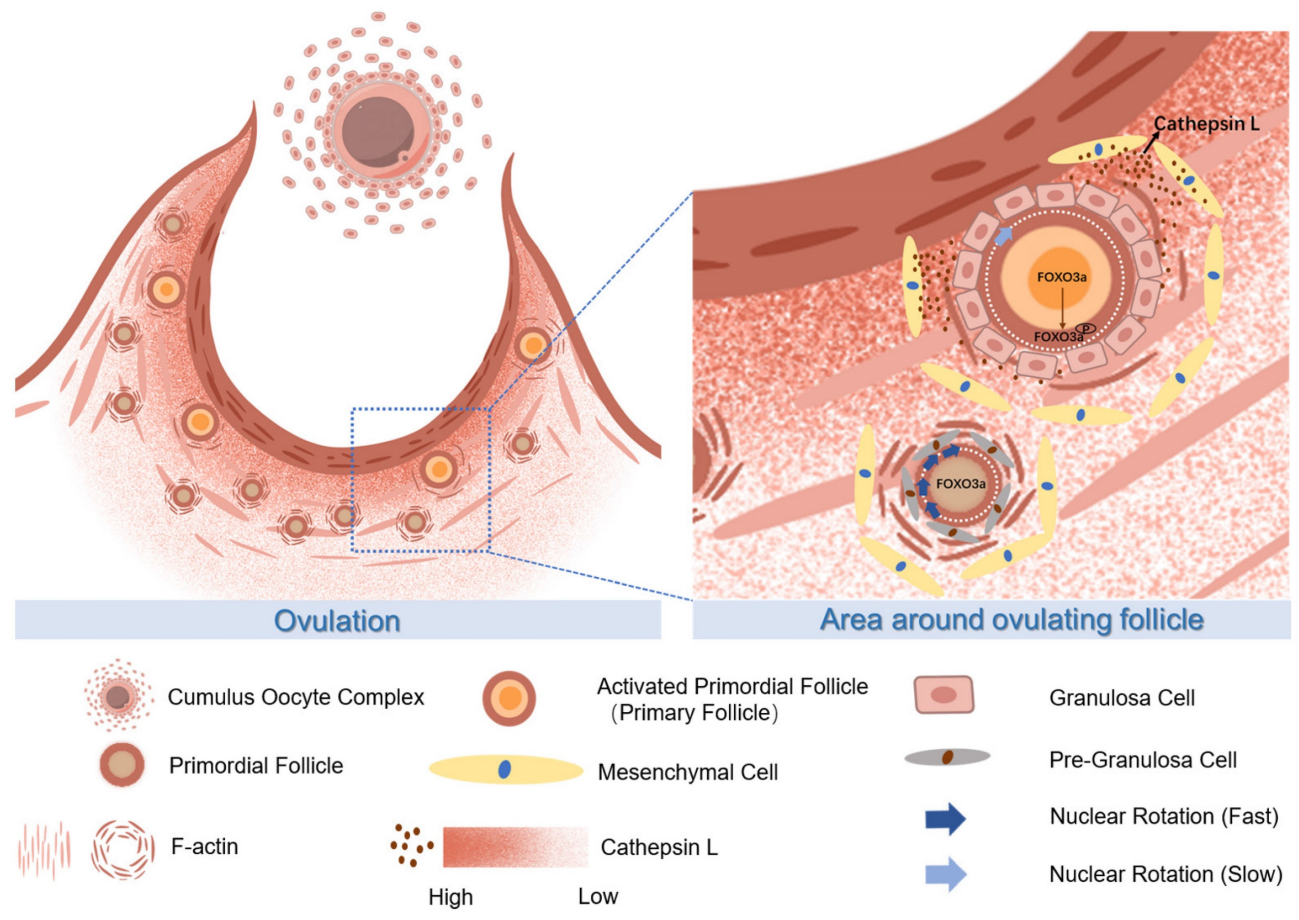
** The proposed molecular mechanisms of CTSL activated primordial follicles adjacent to the ovulatory follicle by degrading extracellular matrix.** During ovulation, the primordial follicle surrounding the large follicle is activated with the release of the mature oocyte. The CTSL secreted by the mesenchymal cells near the ovulating follicle degrades the F-actin of the surrounding primordial follicle, destroys its ECM structure, reduces the mechanical stress on it, and causes the rotation rate of the primordial follicle nucleus to slow down and eventually become activated.

This study uncovers a novel molecular relationship between ovulatory follicles and the orderly activation of adjacent PmFs, two crucial phenomena in female reproduction. Cyclic ovulation plays a pivotal role in activating PmFs near ovulatory follicles in multi-ovulatory mice, reflecting the intricate balance of fertility maintenance *in vivo* (Figure 7). Signals from ovulating follicles stimulate the surrounding non-growing follicles, initiating their growth in each cycle. Multiple mechanisms likely drive PmF recruitment *in vivo*. The initial wave of PmF activation occurs in the fetal ovaries of mice, even in the absence of growing follicles, contributing to preantral follicle development in juvenile mice. According to Dai *et al*., these PmFs are activated by oocyte-derived signals rather than preGCs signals 2. In adult mice, some PmFs within the defined zone of preovulatory follicles may not be recruited. Interestingly, the activation of PmFs within an ovary is not randomly selected in each cycle but rather depends on their proximity to ovulatory follicles. Ovulation leads to significant ECM degradation and remodeling within a 40 μm range around the follicle. The gradient of enzymes, particularly the CTSL protein secreted by peri-ovulatory follicle mesenchymal cells, likely activates surrounding PmFs by relieving ECM-associated mechanical stress. Consequently, PmFs closer to the ovulatory follicle are more likely to be activated first. This sequential process of ovulation followed by PmF activation is crucial for maintaining the PmF reserve.

Numerous studies have independently investigated follicle formation, activation, growth, ovulation, and atresia over the past decades. Research has partially elucidated the mechanisms of PmF formation and activation 2, 15, 44-47. Advances in understanding PmF activation at the molecular level in murine models and through ovarian interventions have enhanced knowledge of female fertility maintenance and improved assisted reproduction techniques for POI patients via IVA procedures 19. Additionally, infertile women have benefited from the understanding of the hypothalamus-pituitary-ovary (HPG) axis and follicular paracrine molecules, which assist in antral follicle development, maturation, and ovulation 48-51. Importantly, the classic negative feedback regulation of the HPG axis, mediated by progesterone from the corpus luteum, reveals a causative relationship between corpus luteum lysis and the initiation of antral follicle growth and ovulation. Dominant antral follicle selection, leading to cyclical ovulation, typically begins with corpus luteum lysis 52-54. Results presented here indicate that events associated with ovulation at the end of follicular development trigger processes that cause PmF activation and the development of replacement follicles. While it remains unclear if this represents a universal rule across species, including mono-ovulatory and multiple-ovulatory species, the findings offer insights into the orderly and selective activation of PmFs under physiological conditions in mammals.

Utilizing spatial transcription sequencing, the study examined molecular differences between mesenchymal cells located near the ovulatory follicles and those farther away. The analysis revealed that the most active physiological process near the ovulation site is the degradation and remodeling of the ECM. Previous research indicates that matrix metalloproteinases from the bloodstream contribute to ECM degradation around the ovulation site. However, this study identifies locally produced enzymes from the mesenchymal cells of ovulatory follicle, particularly CTSL, as pivotal for ECM degradation and reconstruction. CTSL, a cysteine proteinase, is highly abundant and ubiquitously expressed 55. While most CTSL resides in lysosomes or endosomal compartments, a fraction of pro-CTSL is secreted by certain tumors and normal endocrine cells. In the ECM, pro-CTSL is converted to its active form, CTSL, which participates in various proteolytic processes, including ECM degradation 56, 57. Coincidentally, the critical role of CTSL in reproduction is underscored by the observation that *Ctsl* knockout mice are infertile for unknown reasons 58. In medaka fish, CTSL contributes to follicle layer degeneration and degradation post-ovulation through the activation of Plau1 59. Consistent with this, levels of PAI-1 and tPA were elevated during ovulation in this study. Additional support for CTSL's importance during ovulation comes from the fact that its transcription is directly regulated by the progesterone receptor (PR) in preovulatory follicles in response to the LH surge, correlating with transient PR expression in human preovulation follicles 60. In summary, this study suggests that the enriched CTSL surrounding ovulatory follicles in mice ovaries are crucial for ECM degradation. This process is not only essential for ovulation but also for the activation of PmFs.

Increasing evidence highlights the critical role of ovarian ECM in follicle development. The ovarian ECM consists of follicular ECM and stromal ECM 61. This study suggests that ovulation-related activation of nearby PmFs is more likely mediated by the stromal ECM rather than the follicular ECM. The detachment of the COC results from the degradation of the follicular basal lamina and cortical stromal ECM at the follicle apex 62. Additionally, mechanical stress from the stromal ECM in prepubertal human ovaries is higher than in reproductive-age ovaries, creating a stiff niche that is non-permissive to prepubertal follicle activation and growth, thereby maintaining quiescence 63. Collectively, these findings suggest that the stromal ECM significantly contributes to PmF activation in adults.

Releasing the mechanical stress surrounding the PmF is a crucial factor for inducing PmF activation. A recent study found that an intact ECM around PmFs ensures rapid oocyte nuclear rotation, which is closely associated with maintaining the non-growing state of PmFs 26, 64. Consistent with this, PmFs in ovulating ovaries exhibited slower nuclear rotation speeds compared to PmFs in non-hormonally treated ovaries. Additionally, key signaling pathways responsible for PmF activation, including phosphorylated mTOR (p-mTOR), were elevated in the area of ovulatory follicles. The typical translocation of phosphorylated FOXO3a (p-FOXO3a) from the nucleus to the cytoplasm in oocytes was observed in PmFs adjacent to ovulatory follicles compared to controls. However, although Kawamura *et al*. found that ovarian fragmentation increased actin polymerization and disrupted Hippo signaling 21, suggesting Hippo signaling's role in follicle development, this study did not observe changes in Hippo signaling. Therefore, the molecular mechanisms of ovulation-related PmF activation may differ from those involved in ovarian intervention treatments.

In addition to KITL and NGF, CTSL emerges as a novel molecule outside the cellular environment of PmFs that can trigger PmF activation. Further studies are needed to fully explain CTSL-induced PmF activation *in vivo* across multiple species.

In conclusion, this study reveals the pivotal role of ECM degradation by CTSL around an ovulatory follicle. The increased CTSL in the ovulatory follicle niche, induced by the LH surge, not only contributes to ovulation but also activates adjacent PmFs. These findings provide a new perspective for explaining the orderly and selective activation of PmFs in each estrous cycle under physiological conditions in mammals.

## Supplementary Material

Supplementary figures and tables.

Supplementary table 1.

Supplementary table 2.

Supplementary video 1.

Supplementary video 2.

## Abbreviations

COC: cumulus oocyte complexes
mGC: mural granulosa cell
pGC: precursor granulosa cell
GC: granulosa cell
ED: ethynodiol diacetate
ECM: extracellular matrix
tPA: tissue plasminogen activator
CTSL: cathepsin L
dpp: postpartum
PmF: primordial follicle
CSO: consecutive superovulation
CRO: consecutive restrained ovulation
