# Supplementary material for: Participation of preovulatory follicles in the activation of primordial follicles in mouse ovaries
Source: Int J Biol Sci. 2024 Jul 8;20(10):3863–80. doi: 10.7150/ijbs.95020 (PMC11302884; doi:10.7150/ijbs.95020)
Supplement: Supplementary file 1 — Supplementary figures and tables. [file ijbsv20p3863s1.pdf]

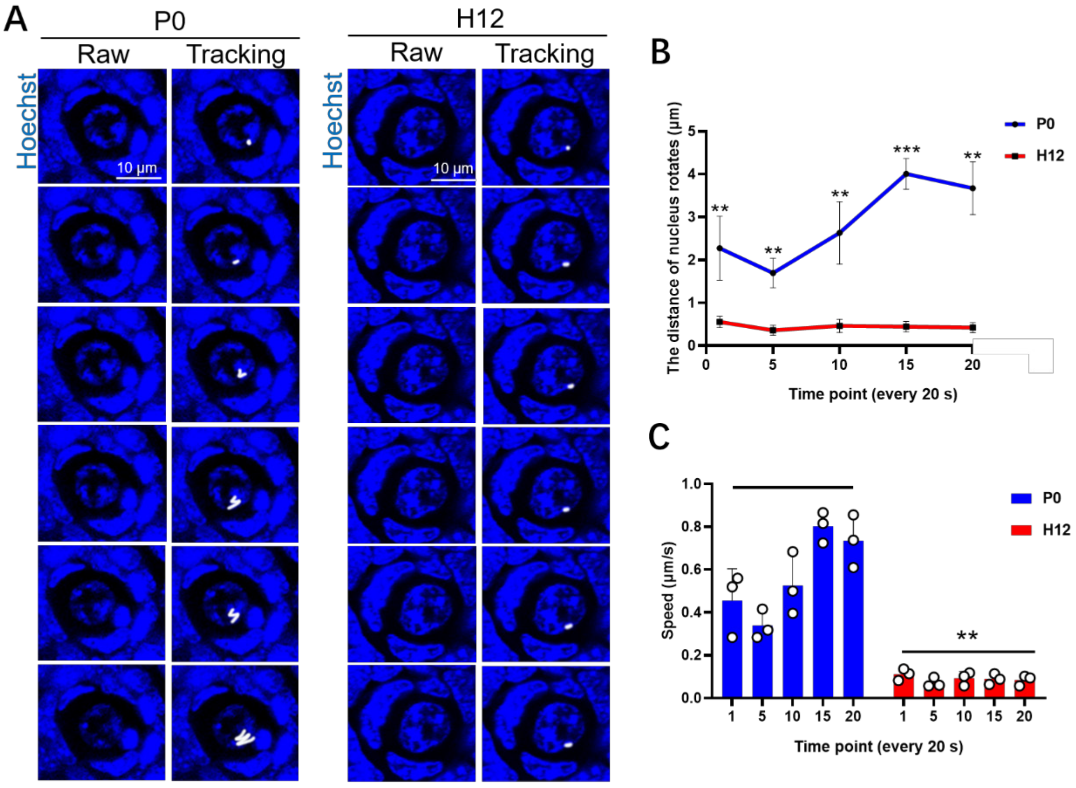

Figure S1. P0 and H12 ovarian PmFs nucleus rotation. (**A**) P0 and H12 ovarian PmFs nucleus rotation. Raw represents the original image. Tracking represents the tracking image, and the solid white line represents the track of the rotation of PmF nucleus. Hoechst (blue). Scale bar = 10 μm. (**B**) Calculate the distance of kernel rotation per unit time based on the tracking data. (**C**) The average rate of nuclear rotation is calculated based on the distance of the core rotation per unit time. The X-axis represents the time point of each image acquisition (every 20 seconds). $*p < 0.05$; $**p < 0.01$; ns $p \geq 0.05$.

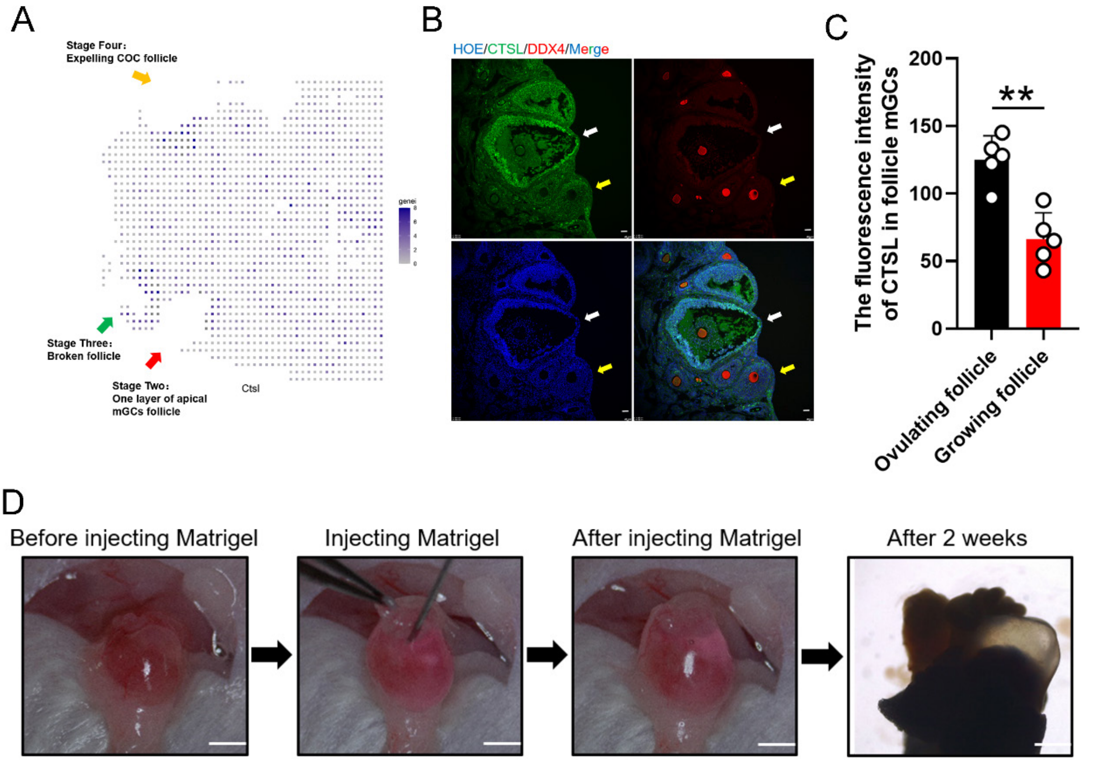

Figure S2. CTSL was highly expressed in the mGCs of ovulating follicles. (**A**) Spatial transcriptome sequencing panel showed the distribution map of the CTSL mRNAs throughout the slide. The darker the color, the higher the mRNA level was recorded. Each pixel represented 20 μm. (**B**) Immunofluorescent staining of CTSL. White arrows indicated ovulating follicle and yellow arrows indicated non-ovulating follicle (Growing follicle). DDX4 (red), CTSL (green), Hoechst (blue). Scale bar = 40 μm. (**C**) The fluorescence intensity of CTSL in follicle in (B) was analyzed. (**D**) Schematic diagram of in situ injection of Matrigel into ovarian bursa. Scale bar = 100 μm.

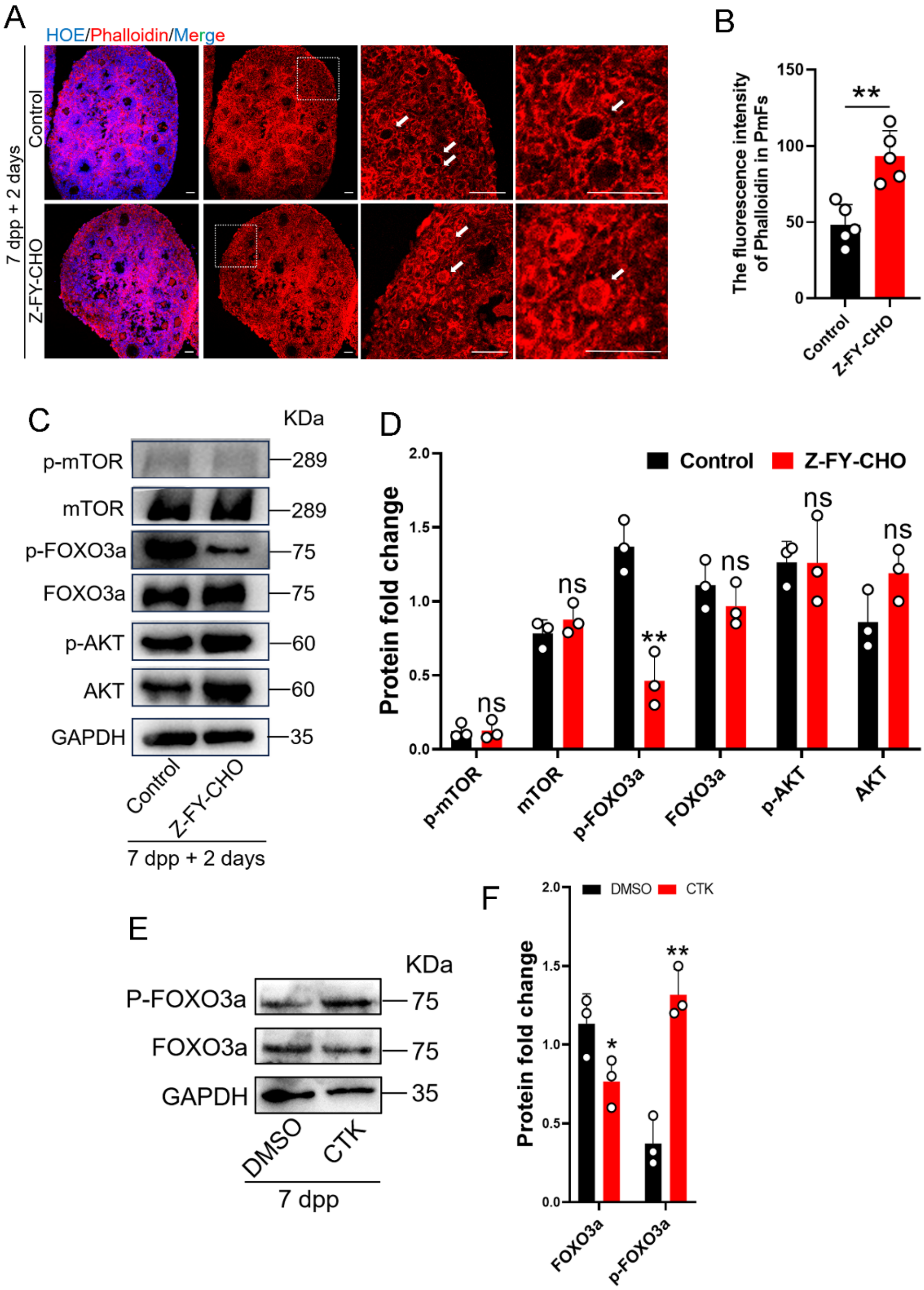

Figure S3. Inhibition of CTSL can slow down the rate of ECM degradation around PmF. (**A**) Immunofluorescent staining of Phalloidin in Control groups and Z-FY-CHO-treated groups. White arrows indicated ovarian cortical PmFs. Phalloidin (red), Hoechst (blue). Scale bar = 40 μm. (**B**) Fluorescence intensity of Phalloidin in cortical PmFs based on (A). (**C**) Western blot analysis of Control ovaries and Z-FY-CHO-treated ovaries on PmF activation related protein levels. (**D**) Quantification of ratio respective proteins normalized to GAPDH in (C). (**E**) The effect of CTK on the protein expression levels of p-FOXO3a and FOXO3a proteins. (**F**) Quantification of ratio respective proteins normalized to GAPDH in (E).
